# Development and Validation of a Nomogram for Predicting Generalization in Patients With Ocular Myasthenia Gravis

**DOI:** 10.3389/fimmu.2022.895007

**Published:** 2022-07-07

**Authors:** Zhe Ruan, Chao Sun, Yanlin Lang, Feng Gao, Rongjing Guo, Quan Xu, Liping Yu, Songdi Wu, Tao Lei, Yu Liu, Min Zhang, Huanhuan Li, Yonglan Tang, Ting Gao, Yanwu Gao, Xiaodan Lu, Zhuyi Li, Ting Chang

**Affiliations:** ^1^ Department of Neurology, Tangdu Hospital, The Fourth Military Medical University, Xi’an, China; ^2^ Department of Neurology, West China Hospital, Sichuan University, Chengdu, China; ^3^ Department of Neuroimmunology, Henan Institute of Medical and Pharmaceutical Sciences, Zhengzhou University, Zhengzhou, China; ^4^ Department of Thoracic Surgery, Jiangxi Provincial People’s Hospital Affiliated to Nanchang University, Nanchang, China; ^5^ Department of Neurology, Xianyang First People’s Hospital, Xianyang, China; ^6^ Department of Neurology, Xi'an No.1 Hospital, Xi’an, China; ^7^ Department of Neurology, Xi’an Fourth People’s Hospital, Xi’an, China

**Keywords:** nomogram, generalization, ocular myasthenia gravis, prediction model, immunotherapy

## Abstract

**Background:**

This study aims to develop and validate a nomogram for predicting 1- and 2-year generalization probabilities in patients with ocular myasthenia gravis (OMG).

**Methods:**

In total, 501 eligible patients with OMG treated at seven tertiary hospitals in China between January 2015 and May 2019 were included. The primary outcome measure was disease generalization. A nomogram for predicting 1- and 2-year generalization probabilities was constructed using a stepwise Cox regression model. Nomogram performance was quantified using C-indexes and calibration curves. Two-year cumulative generalization rates were analyzed using the Kaplan−Meier method for distinct nomogram-stratified risk groups. The clinical usefulness of the nomogram was evaluated using decision curve analysis (DCA).

**Result:**

The eligible patients were randomly divided into a development cohort (n=351, 70%) and a validation cohort (n=150, 30%). The final model included five variables: sex, onset age, repetitive nerve stimulation findings, acetylcholine receptor antibody test results, and thymic status. The model demonstrated good discrimination (C-indexes of 0.733 and 0.788 in the development and validation cohorts, respectively) and calibration, with good agreement between actual and nomogram-estimated generalization probabilities. Kaplan−Meier curves revealed higher 2-year cumulative generalization rates in the high-risk group than that in the low-risk group. DCA demonstrated a higher net benefit of nomogram-assisted decisions compared to treatment of all patients or none.

**Conclusion:**

The nomogram model can predict 1- and 2-year generalization probabilities in patients with OMG and stratified these patients into distinct generalization risk groups. The nomogram has potential to aid neurologists in selecting suitable patients for initiating immunotherapy and for enrolment in clinical trials of risk-modifying treatments.

## Introduction

Myasthenia gravis (MG) is an acquired autoimmune disorder that affects the postsynaptic membrane at the neuromuscular junction (NMJ). The predominant manifestation of MG is weakness of the ocular, bulbar, respiratory, and limb muscles. Ocular muscles are easily affected with typical symptoms being diplopia and ptosis. In the majority of patients with MG (50-85%), disease starts with isolated ocular symptom, referred to as ocular MG (OMG) (1). However, 30-80% of patients develop systemic muscular weakness converting to generalized myasthenia gravis (GMG), typically within the first 1 to 2 years from ocular symptoms onset (2, 3). Once the condition becomes generalized, patients experience more severe symptoms, and even life-threatening when respiratory weakness occurrence. Thus, preventing progression from ocular MG to generalized MG is crucial. In this regard, several studies have reported that early immunotherapy and thymectomy reduced the risk of generalization (4–12).

Nevertheless, long-term immunotherapy especially steroid treatment, is associated with several adverse effects, including avascular bone necrosis, peptic ulcer disease, diabetes, hypertension, osteoporosis, mood disorders, weight gain, glaucoma, cataracts, and opportunistic infections (13). Notably, the risk of generalization differs among patients with OMG: approximately 36-70% of patients do not develop generalization, irrespective of immunotherapy (8, 14, 15). False positives (good responders for thymectomy) may exist due to factors such as over-zealous use of thymectomy and misdiagnosis in retrospective analysis. Individuals who received a thymectomy but would have never progressed to GMG irrespective of the thymectomy (16). An early decision to start immunotherapy or thymectomy may expose this group of patients unnecessarily to the adverse effects of these therapies. Additionally, reports suggest that the generalization rate is substantially lower in the Eastern population than that in Western population (17-23% vs 50-70%) (14, 15, 17, 18). Thus, it is critical to perform early screening of patients at high risk of generalization and to initiate risk-modifying treatments accordingly.

Previous studies have identified various predictive risk factors for generalization, including late onset, abnormal repetitive nerve stimulation (RNS) findings, seropositivity for acetylcholine receptor antibody (AChR-Ab), and the presence of thymoma (14, 17, 19–26). However, there has been a lack of validated instrument to incorporate these risk factors into an individualized prediction of generalization.

In this study, we aimed to develop and validate a nomogram as an easily applicable prognostic model for predicting the risk of generalization in patients with OMG and for stratifying patients into “low” or “high” risk generalization.

## Materials and Methods

### Study Population

**Figure 1:**
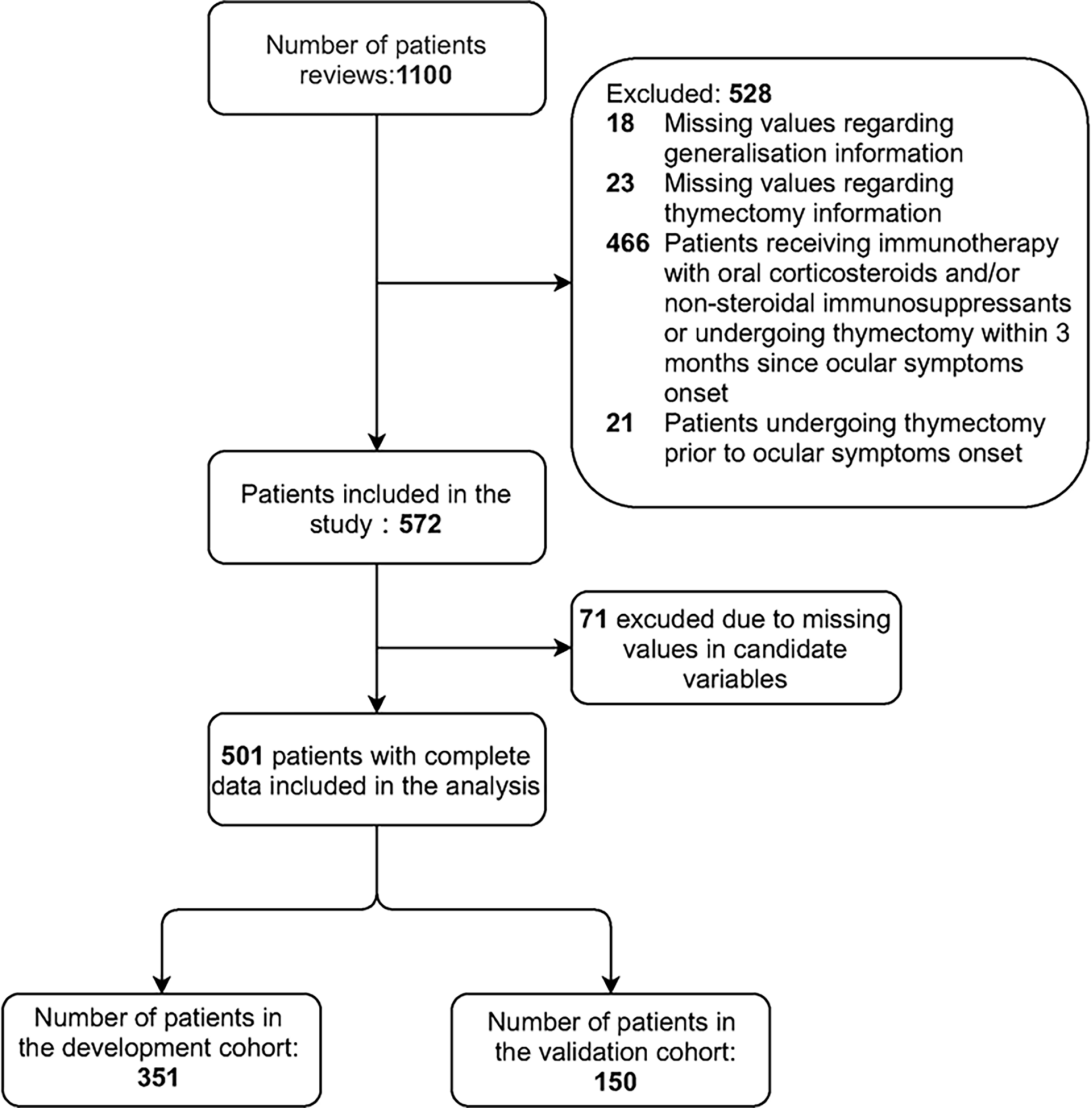
Flowchart of participants included in the study.

We developed and validated a predictive model in a multicenter cohort study of patients with OMG in China between January 2015 and May 2019. The data of all consecutive patients with OMG at onset were retrospectively collected from seven tertiary hospitals. Participants who fulfilled the following criteria were included: 1) diagnosis of OMG and 2) minimum 3-month isolated extraocular symptoms at onset. In all participating centers, chest imaging tests (either computed tomography [CT] or magnetic resonance imaging [MRI] scans) were routinely performed, and thymic status (e.g., normal, thymic hyperplasia, or thymoma) was evaluated radiologically and pathologically. Participants with one or more of the following conditions were excluded: 1) missing values regarding generalization information (generalization occurrence or generalization time); 2) missing values regarding thymectomy information (thymectomy performance or surgery time); 3) patients receiving immunotherapy with oral corticosteroids and/or non-steroidal immunosuppressants (e.g., azathioprine, mycophenolate mofetil, and tacrolimus) or those undergoing thymectomy within 3 months after ocular symptoms onset; and 4) patients undergoing thymectomy prior to ocular symptom onset. The selection process is illustrated in **Figure 1**.

The clinical data of the participants were extracted from the MG database of Tangdu Hospital, which was created in 2019, and electronic records from other medical centers based on the uniform items. Most eligible patients had a face-to-face interview at 1-3-month intervals and were estimated routinely through Quantitative Myasthenia Gravis score (QMGs), MG-Activities of Daily Living (MG-ADL), and MG-Composite (MGC) scores. For patients who failed to face-to-face interview were followed up by telephone interview. The symptoms and medication data were recorded, and MG-ADL was also collected. Ten neurologists and three nurses participated in data collection. The diagnosis of OMG was confirmed by at least two neurologists. Research data have been used in previously published articles (19).

The study was approved by the Ethics Committee of Tangdu Hospital, the Fourth Military Medical University (K202012-02). Research conducted in other medical centers was also approved by the respective ethics committees (**Table S1**). The requirement for written informed consent was waived by the ethics committee due to the retrospective nature of the study.

### Data Collection

Based on the results of our previous research and a literature search, we collected the following predictive factors of generalization: sex (male or female) (20), onset age (21–24), onset symptoms (ptosis and diplopia) (21), RNS findings (normal or abnormal) (17), AChR-Ab test results (seropositive or seronegative) (14, 17, 25), autoimmune comorbidities (e.g., systemic lupus erythematosus, multiple sclerosis, rheumatoid arthritis, Hashimoto’s thyroiditis, and optic neuromyelitis spectrum disease), and thymic status (normal, thymic hyperplasia, or thymoma) (14, 26). RNS was performed on the facial, ulnar, axillary and accessory nerves at a frequency of 3 Hz, and recorded on the orbicularis oculi, abductor digiti minimi, deltoid and trapezius muscles. A decrement of >10% from the first to the fourth compound muscle action potential (CMAP) is regarded as abnormal. For patients with multiple RNS tests, only the earliest measurement was recorded in the database. AChR-Ab was detected by commercial enzyme-linked immunosorbent assay (ELISA) or radioimmunoassay in Wuhan kangshengda Medical Laboratory and Henan Institute of Medical and Pharmaceutical Sciences in China. A cut-off value of 0.4 nmol/L was treated as abnormal for ELISA test, and 0.5 nmol/L treated as abnormal for radioimmunoassay test. All patients routinely performed MuSK antibody tests by cell-based assay (CBA). Preoperative radiological and postoperative pathological findings were used to evaluate thymic status. For patients who underwent thymectomy, evaluation depended on pathological findings. Whereas, for patients who did not undergo thymectomy, evaluation depended on radiological findings (such as CT, enhanced CT and MRI scans). Patients with any missing values for these variables were excluded from subsequent analyses.

### Outcome Measure and Follow-Up

The primary outcome measure was disease generalization, defined as the appearance of symptoms or signs that were not limited to the extraocular muscles (i.e., weakness of the facial, bulbar, neck, limb, or respiratory muscles) (27). Disease generalization was evaluated by a neuromuscular specialist who interviewed patients for their recent history of MG-related symptoms and assessed the distribution of weakness through QMGs, MG-ADL, MGC scores. Follow-up commenced from ocular symptom onset and ceased at confirmation of generalization, or immunotherapy initiation, or thymectomy performance, or final follow-up assessment defined as 60 months.

### Statistical Analysis

All analyses were performed with R 4.0.3 (The R Foundation for Statistical Computing, Vienna, Austria). For the development of prediction model, we used “pmsampsize” package in R version to estimate sample size (28, 29). This package proposed by Riley et al. is the standard to estimate minimum sample size in developing a new multivariable prediction model. For time-to-event outcome, following criteria should be required: 1) Small overfitting defined by an expected shrinkage of predictor effects by 10% or less; 2) Small absolute difference of 0.05 in the model’s apparent and adjusted Nagelkerke’s R-squared value; 3) Precise estimation (within +/- 0.05) of the average outcome risk in the population for a key timepoint of interest for prediction. Based on the previous results in our study (19, 30), the parameters for calculating sample size are as follows: Cox Snell R-squared: 0.2; prediction time point: 2 years; the average follow-up time: 2.1 years; the proportion of total outcome: 0.23; the number of candidate predictor parameters: 8. The calculated sample size was 319. To provide adequate test efficiency, we included 501 eligible patients with 351 in the development cohort, which is much higher than the estimated sample size.

The *X* ^2^ test or Fisher exact test was used for comparison of categorical variables and the student’s *t-*test or Mann−Whitney U test was used for comparison of continuous variables between the development and validation cohorts. Multivariable Cox proportional regression analysis was used to calculate the hazard ratios (HRs) and 95% confidence intervals (CIs) of the predictive variables and to construct the nomogram.

All candidate variables were included in a Cox proportional hazards regression analysis and passed the proportional hazard model hypothesis test. The full Cox model was simplified with stepwise regression using the “step” function with “both”, as per the R package “stats”, and the likelihood ratio test with Akaike’s information criterion was used as the stopping rule (31).

Based on the multivariable Cox analysis, a nomogram for the estimation of 1- and 2-year generalization probabilities was formulated using “rms” package in R version in the development cohort (32). The discrimination ability of the nomogram was measured using the Harrell concordance index (C-index). Consistency between actual and nomogram-predicted generalization probabilities was assessed using calibration curves (1,000 resampling bootstrap) in both the development and validation cohorts (33).

Receiver operating characteristic (ROC) curves were plotted to determine the optimal cut-off value, which was calculated using Youden’s index (sensitivity + specificity - 1) (34). Patients were stratified into low- and high-risk generalization groups according to the optimal cut-off value. Two-year cumulative generalization rates were analyzed using the Kaplan−Meier method in the development and validation cohorts and compared using the log-rank test. The clinical usefulness of the nomogram was evaluated using decision curve analysis (DCA) by quantifying the net benefits at different threshold probabilities in the development and validation cohorts (35). Statistical significance was set at *P* < 0.05. All statistical tests were two-tailed.

## Results

### Study Population

A total of 71 patients in our cohort had missing data. The missing variables included the results of RNS, AChR-Ab and thymic status. The pattern of missing data is shown in **Figure S1**. 501 eligible patients were included in the final analysis, comprising 351 patients in the development cohort and 150 patients in the validation cohort. In the development cohort, the mean (SD) onset age was 44.7 (19.5) years, and 163 (46.4%) were women. During the follow-up period, 83 (23.6%) patients developed generalization. A median follow-up time was 17.0 (IQR: 7.0-48.0) months. In the validation cohort, the mean (SD) onset age was 44.3 (20.8) years, and 76 (50.7%) were women. Of these patients, 35 (23.3%) developed generalization and a median follow-up time was 12.5 (IQR: 7.3-36) months. No significant differences were observed in sex, mean onset age, onset symptoms, RNS findings, AChR-Ab test results, and thymic status between the development and validation cohorts. **Table 1** presents a detailed comparison of the demographics and predictive variables between the development and validation cohorts.

**Table 1:** Baseline characteristics of the development and validation cohorts.

	Overall (n=501)	Development cohort (n=351)	Validation cohort (n=150)	*P*-value
**Sex, No. (%)**				0.44
Male	262 (52.3)	188 (53.6)	74 (49.3)	
Female	239 (47.7)	163 (46.4)	76 (50.7)	
**Onset age, mean (SD), y**	44.6 (19.9)	44.7 (19.5)	44.3 (20.8)	0.82
**Autoimmune comorbidities, No. (%)** ^a^				0.22^b^
No	488 (97.4)	344 (98.0)	144 (96.0)	
Yes	13 (2.6)	7 (2.0)	6 (4.0)	
**Onset symptoms, No. (%)**				0.99
Ptosis	310 (61.9)	217 (61.8)	93 (62.0)	
Diplopia/diplopia and ptosis	191 (38.1)	134 (38.2)	57 (38.0)	
**RNS findings, No. (%)**				0.24
Normal	252 (50.3)	170 (48.4)	82 (54.7)	
Abnormal	249 (49.7)	181 (51.6)	68 (45.3)	
**AChR-Ab, No. (%)**				0.51
Seronegative	132 (26.3)	89 (25.4)	43 (28.7)	
Seropositive	369 (73.7)	262 (74.6)	107 (71.3)	
**Thymic status, No. (%)**				0.93
Normal	322 (64.3)	226 (64.4)	96 (64.0)	
Thymic hyperplasia	100 (20.0)	71 (20.2)	29 (19.2)	
Thymoma	79 (15.8)	54 (15.4)	25 (16.7)	
**Neostigmine test, No. (%)**				0.99^b^
Negative	18 (3.8)	13 (3.9)	5 (3.5)	
Positive	460 (96.2)	323 (96.1)	137 (96.5)	
**Follow-up time, median (IQR), m**	15.0 [7.0,48.0]	17.0 [7.0,48.0]	12.5 [7.3,36.0]	0.60^c^
**Generalization, No. (%)**	118 (23.6)	83 (23.6)	35 (23.3)	0.99

SD, standard deviation; IQR, interquartile range; RNS, repetitive nerve stimulation; AChR-Ab, acetylcholine receptor antibody; No., number.

a Autoimmune comorbidities included systemic lupus erythematosus, multiple sclerosis, rheumatoid arthritis, Hashimoto’s thyroiditis, and optic neuromyelitis spectrum disease.

b Fisher exact test.

c Mann−Whitney U test.

### Model Development and Validation

Using stepwise selection in the Cox proportional hazards regression model, five predictive variables (sex, onset age, RNS findings, AChR-Ab, and thymic status) were retained in the final simplified model and were used to construct the nomogram. In the development cohort, multivariable Cox proportional regression analysis demonstrated that late-onset age (adjusted HR, 1.02; 95% CI, 1.01-1.04; *p*<0.001), abnormal RNS findings (adjusted HR, 2.80; 95% CI, 1.67-4.70; *p*<0.001), and presence of thymoma (adjusted HR, 1.84; 95% CI, 1.10 -3.07; *p* = 0.02) were independently associated with an increased risk of generalization. Similar associations were observed in the validation cohort. **Table 2** presents the associated hazard ratios of the predictive variables in the development and validation cohorts.

**Table 2:** Multivariable HRs for association between predictive variables and generalization.

Predictive variables	Development cohort		Validation cohort	
	HR (95% CI)	*P*-Value	HR (95% CI)	*P*-value
**Sex**				
Male	1		1	
Female	1.45 (0.94-2.24)	0.10	1.27 (0.62-2.62)	0.52
**Onset age**	1.02 (1.01-1.04)	<0.001	1.05 (1.02-1.07)	<0.001
**RNS findings**				
Normal	1		1	
Abnormal	2.80 (1.67-4.70)	<0.001	5.44 (2.33-12.70)	<0.001
**AChR-Ab**				
Seronegative	1		1	
Seropositive	2.42 (1.00-5.88)	0.05	3.27 (0.97-10.96)	0.06
**Thymic status**				
Normal	1		1	
Thymic hyperplasia	1.52 (0.83-2.79)	0.17	1.33 (0.49-3.58)	0.57
Thymoma	1.84 (1.10-3.07)	0.02	2.72 (1.20-6.62)	0.03

RNS, repetitive nerve stimulation; AChR-Ab, acetylcholine receptor antibody; m, month.

**Figure 2:**
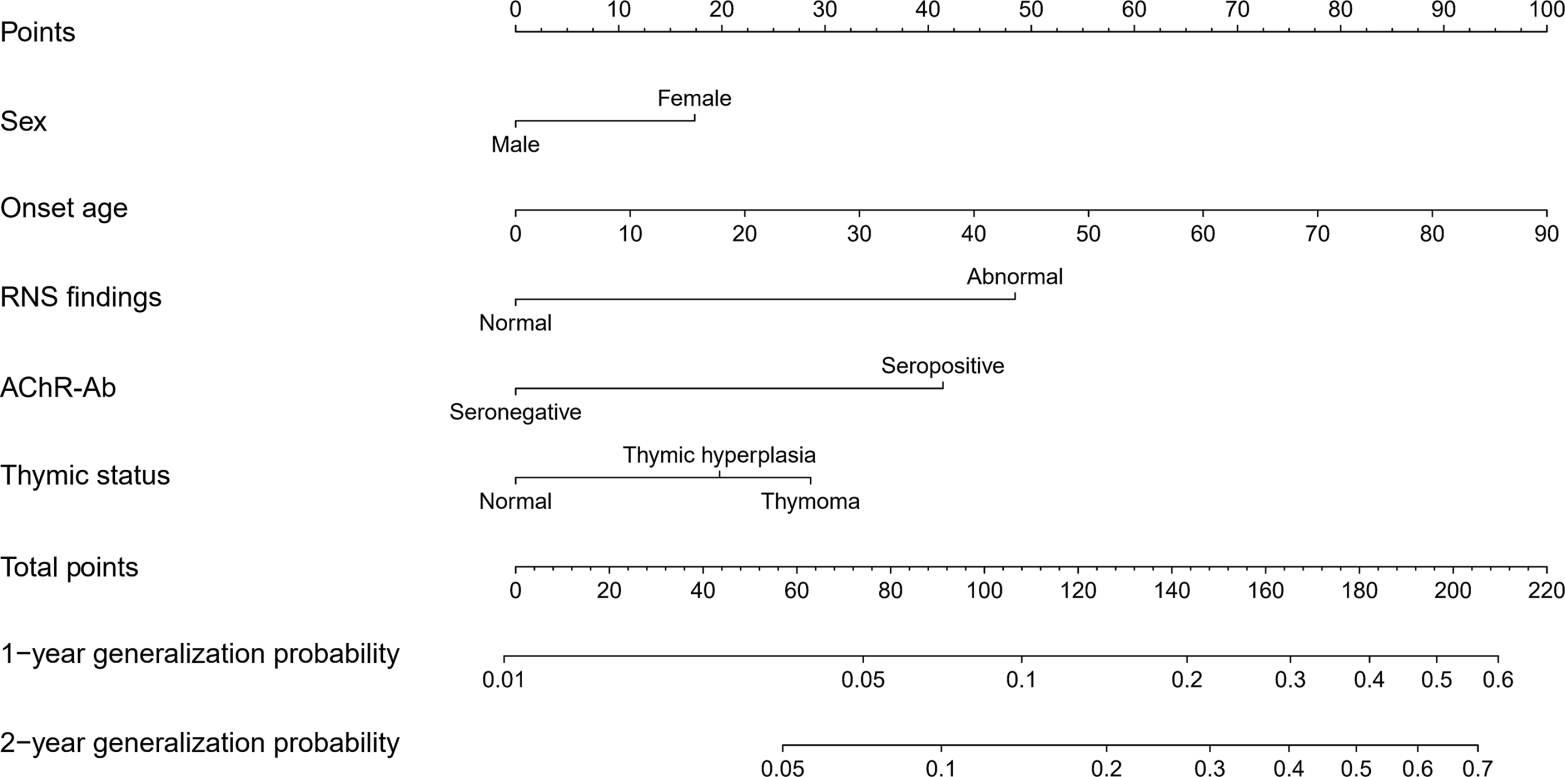
Nomogram predicting the 1- and 2-year generalization probabilities of patients with OMG. The nomogram summed the points identified on the scale for each variable. The total points projected on the bottom scales indicate the 1- and 2-year generalization probabilities.

The nomogram for predicting 1- and 2-year generalization probabilities is presented in **Figure 2**. The nomogram is based on proportionally converting each regression coefficient in Multivariable Cox analysis to a 0‐ to 100‐point scale. Within those five variables that construct the nomogram, each covariate was assigned a score by drawing corresponding vertical line straight down to the axis labeled points. By summing the total score and locating it on the total points scale, the individual probabilities of 1- or 2-year generalization can be determined. To make the model more user-friendly, we provide a freely available web calculator (https://doctorchang.shinyapps.io/OMG_generalization2/). This web-based tool can be used for more precise calculations and also reports 95% prediction intervals; the assumed endpoint is adjustable up to 60 months.

**Figure 3:**
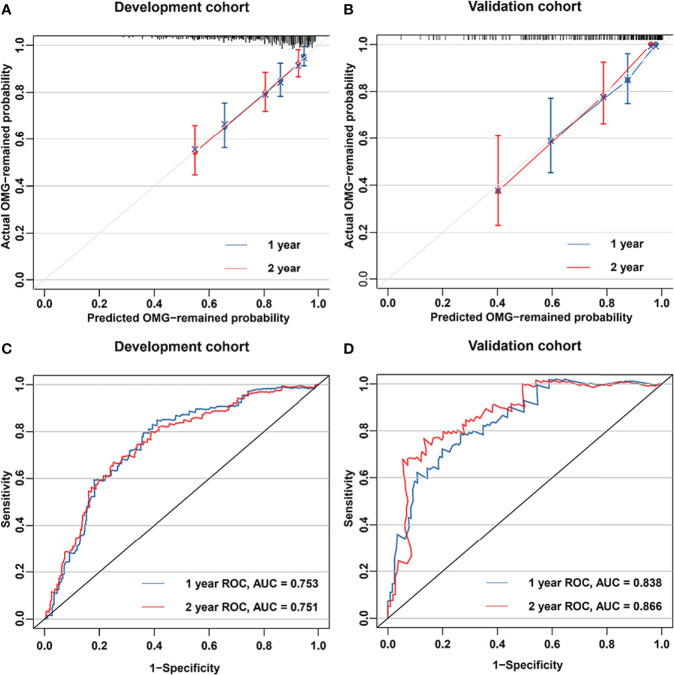
Nomogram performance. Calibration plots for estimating 1- and 2-year generalization probabilities are presented for the development cohort **(A)** and validation cohort **(B)**. The x- and y-axes represent the nomogram-predicted and actual generalization probabilities, respectively. The 45° grey line is the reference line indicating a perfect calibration. The blue and red lines represent the nomogram-predicted 1- and 2-year generalization probabilities, respectively. Areas under the curves of the model for predicting 1- and 2-year generalization probabilities in the development cohort **(C)** and validation cohort **(D)**. The red and blue lines represent the nomogram-predicted 1- and 2-year generalization probabilities, respectively.

Moreover, C-index and Calibration curve were generated to evaluate the performance of the nomogram. The model exhibited a C-index of 0.733 (95% CI, 0.676-0.790) for the development cohort and 0.788 (95% CI, 0.710-0.865) for the validation cohort. Calibration curves with 1,000 resampling bootstraps demonstrated good agreement between the nomogram-predicted and actual generalization probabilities in both the development and validation cohorts (**Figures 3A, B**).

### Comparison of Clinical Characteristics Between Nomogram-Stratified Risk Groups

**Figure 4:**
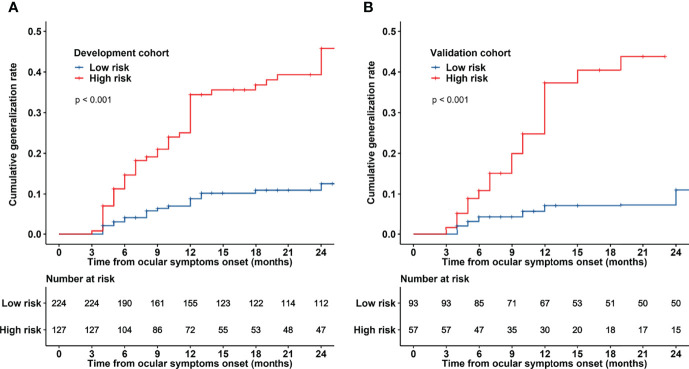
Kaplan−Meier curves for patients with OMG based on risk group stratification in the development and validation cohorts. Patients were stratified into low- and high-risk generalization groups based on the optimal cut-off value (26%). Two-year cumulative generalization rates are presented for the development cohort **(A)** and validation cohort **(B)**.

ROC curves of the model predicting generalization probabilities in the development and validation cohorts are presented in **Figures 3C, D**. The areas under the curve (AUCs) of the model predicting 1- and 2-year generalization probabilities were 0.753 and 0.751, respectively, in the development cohort and 0.838 and 0.866, respectively, in the validation cohort. The optimal 2-year cut-off value of ROC curve in the development cohort was determined to be 0.26. The patients were subsequently stratified into two risk groups based on the optimal cut-off value: a low-risk generalization group (predicted 2-year generalization probability ≤ 0.26) and a high-risk generalization group (predicted 2-year generalization probability > 0.26). The comparison of clinical characteristics between the two risk groups is presented in **Table 3**. Significant differences were observed in onset age, RNS findings, AChR-Ab, and thymic status between different risk groups in the development and validation cohorts. Patients in the high-risk group had an older onset age, higher proportion of abnormal RNS findings, seropositivity for AChR-Ab, and concurrent thymoma. The Kaplan−Meier curve demonstrated that the 2-year cumulative generalization rate was significantly higher in the high-risk group than in the low-risk group both in the development and validation cohorts (**Figures 4A, B**).

**Table 3:** Comparison of clinical characteristics between the low- and high-risk groups in the development and validation cohorts.

	Development cohort (n=351)		*P*-Value	Validation cohort (n=150)		*P*-Value
	Low-risk (n=224)	High-risk (n=127)		Low-risk (n=93)	High-risk (n=57)	
**Sex, No. (%)**			0.02			0.42
Male	131 (58.5)	57 (44.9)		43 (46.2)	31 (54.4)	
Female	93 (41.5)	70 (55.1)		50 (53.8)	26 (45.6)	
**Onset age, mean (SD), y**	39.3 (20.3)	54.2 (13.7)	<0.001	37.7 (21.3)	55.0 (14.6)	<0.001
**RNS findings, No. (%)**			<0.001^a^			<0.001^a^
Normal	162 (72.3)	8 (6.3)		73 (78.5)	9 (15.8)	
Abnormal	62 (27.7)	119 (93.7)		20 (21.5)	48 (84.2)	
**AChR-Ab, No. (%)**			<0.001^a^			<0.001^a^
Seronegative	89 (39.7)	0 (0.0)		41 (44.1)	2 (3.5)	
Seropositive	135 (60.3)	127 (100.0)		52 (55.9)	55 (96.5)	
**Thymic status, No. (%)**			<0.001^a^			<0.007^a^
Normal	163 (72.8)	63 (49.6)		67 (72.0)	29 (50.9)	
Thymic hyperplasia	48 (21.4)	23 (18.1)		17 (18.3)	12 (21.1)	
Thymoma	13 (5.8)	41 (32.3)		9 (9.7)	16 (28.1)	
**Follow-up time, median (IQR), m**	23.50 [8.00, 60.00]	12.00 [7.00, 31.00]	0.003^b^	24.00 [10.00, 49.00]	12.00 [6.00, 24.00]	0.001^b^

SD, standard deviation; IQR, interquartile range; RNS, repetitive nerve stimulation; AChR-Ab, acetylcholine receptor antibody; No., number.

a Fisher exact test.

b Mann-Whitney U test.

### Decision Curve Analysis

**Figure 5:**
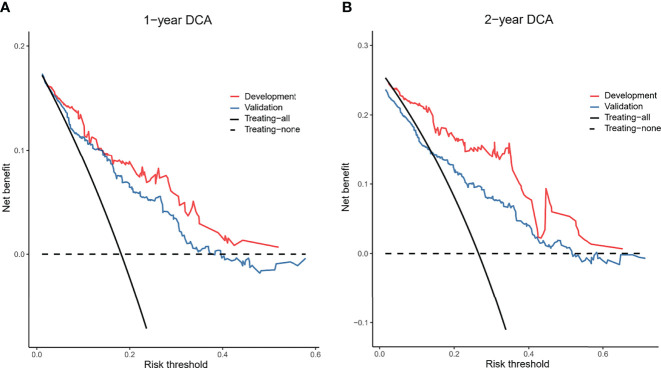
Decision curves of the nomogram model. In the figure, the abscissa is the threshold probability, and the ordinate is the net benefit rate. The horizontal image indicates net benefit when all patients with OMG are considered have not developing generalization and not treated. The oblique image indicates net benefit when all patients with OMG are considered having develop generalization and treated. **(A, B)** depict the decision curves predicting the 1- and 2-year generalization probabilities in the development and validation cohorts, respectively.

The 1- and 2-year net benefits of the nomogram model for the development and validation cohorts are presented in **Figure 5**. Applying the model to the validation cohort, the blue line (corresponding to the model) clearly has higher net benefit across a wide range of risk thresholds (15-50%) shown in **Figure 5**. For instance, at the 30% risk threshold, the net benefit was 10% in the nomogram model. That is, nomogram-assisted decisions of starting treatment in high-risk patients yield higher benefit compared to treatment of all patients or none.

## Discussion

Based on a large Chinese cohort to date, this study developed and validated a novel nomogram model for predicting 1- and 2-year probabilities of generalization in immunotherapy-naïve patients with OMG. Four key findings were disclosed in this study (1): The natural generalization rate at the median follow-up (15.0 months) was 23.6% in patients with OMG; (2) in multivariable analysis, late-onset age, abnormal RNS findings and presence of thymoma were independently associated with an increased risk of generalization; (3) the nomogram model have good predictive power and ability of generalization risk stratification; and (4) the nomogram model and free web-based tool have great potential in helping guide treatment decisions.

The nomogram model exhibited good discriminative power (C-indexes of 0.733 and 0.786 in the development and validation cohorts, respectively). Furthermore, the calibration plots demonstrated that the predicted 1- and 2-year generalization probabilities closely corresponded to the actual generalization probabilities in both the development and validation cohorts. The predictive power of this nomogram model was subsequently verified by the 2-year cumulative generalization rates in patients with distinct risk groups stratified using this model. Importantly, these two risk groups demonstrated significant difference in generalization occurrence. The 2-year cumulative generalization rate was significantly lower in the low-risk group than in the high-risk group.

Further comparison of clinical characteristics between the two distinct risk groups revealed that patients in the high-risk generalization group had an older onset age, higher proportion of abnormal RNS findings, seropositivity for AChR-Ab, and concurrent thymoma. Previous studies have indicated that patients with the aforementioned risk factors have a higher generalization risk than that without these risk factors. For example, Wang et al. reported that the age of disease onset was significantly older in the second generalization group (21). Feng et al. also reported that converted patients with OMG had an older onset age (threshold: 43 years) (22). Several studies reported seropositivity for AChR-Ab was a predictor of disease generalization (8, 14, 15, 17, 22, 23, 25, 36–38). Moreover, Peeler *et al.* reported that high AChR-Ab levels were associated with progression from OMG to generalized disease (25). Concurrent thymoma was regarded as a risk factor for generalized disease in both our and other studies (19–26). These results, combined with discrimination and calibration, support the reliability of this nomogram model.

Nevertheless, the clinical consequences of a particular level of discrimination cannot be captured by risk prediction performance, discrimination, and calibration. Clinical usefulness is a key indicator to evaluate the applicability of a prediction model in clinical settings and benefits to patients. Thus, to justify the clinical usefulness of the model, we assessed whether nomogram-assisted decisions would improve patient outcomes using DCA. The result showed both in the development cohort or validation cohort, nomogram-assisted decisions yielded higher net benefit across a wide range of risk thresholds (15-50%) compared to treatment of all patients or none.

Notably, the generalization rate was lower in our study compared with that reported in other studies (3, 4, 6, 8). The discrepancy may be related to the difference in inclusion criteria, censored data and genetic background. Firstly, different criteria previously were set for the diagnosis of OMG with respect to the duration of disease. For example, Grob included only the patients whose disease remained ocular for the first 1 month after onset (3), while Sommer and Oosterhuis required a period of at least 3 months after onset for the diagnosis of OMG (8, 39). Interestingly, Sommer reported a much lower rate of secondary generalization (31%) compared to that reported by Grob (66%) (3). In the present study, we included the patients with a minimum of 3 months for purely ocular symptoms, and the generalization rate was similar to that reported by Sommer. If we included patients who generalized within 3 months, the total generalization rate would be 52.8% (428/811). We set 3-month as a time limit due to the criterion suggested by Oosterhuis that a minimum of 3 months as the limit for purely ocular symptoms before classifying a patient as having OMG. It was claimed that the patients who develop GMG very early may be different from other patients with OMG. Several studies also followed the criterion of Oosterhuis (10, 19, 30).

Secondly, patients were censored at immunotherapy initiation and thymectomy in our study. Such design can eliminate the confounding factors derived from thymectomy and immunotherapy, and fully utilize the censored data, but also lead to data missing regarding generalization in patients who were censored (we were not able to observe their outcome events). This may explain low generalization rate in our study. Two-year generalization rate will be higher (65.9%) if the impact of censored data and duration time of ocular symptoms were not taken into consideration.

Thirdly, the genetic background in Asian might explain lower generalization rate. TEO reported a low generalization rate of OMG to GMG in an Asian population in Singapore. The generalization rate at the median follow-up (40.8 months) was 10.6% (95% CI 7.9-13.3%) and at 2 years of follow-up was 7.7% (95% CI 5.6-9.8%) (17). Secondary generalization developed in 47 (23.3%) of the 202 study subjects, mostly within the first 6 months after symptom presentation in a study conducted in Korean MG patient population (14).

In addition to its application in clinical practice for personalized treatment according to predictive generalization probability, we anticipate that this model can be used as a research tool to select suitable patients for enrolment in randomized controlled trials of risk-modifying treatments. Risk-modifying treatments are becoming increasingly available for MG; however, many MG trials excluded patients with OMG. A major reason for this is that OMG is considered non-severe, and exposure to the risks of immunotherapy is not justified. In this regard, the decision to administer immunotherapy is only justified if the patient demonstrates clear evidence of generalized MG. In addition, as part of the natural history of disease, 36-70% of patients with OMG can achieve spontaneous, long-lasting remission without immunotherapy (8, 14, 15). An early decision to start immunotherapy will increase the potential adverse effects of treatment in these patients without definite benefits. We propose the use of this model for identifying this subset of patients in the future. However, prior to employing this model for personalized treatment and/or clinical trials, external validation and improved prediction by including biomarkers such as genetic and serological factors are required.

The present study has several limitations. First, the retrospective nature of the study may have introduced the risk of bias in the data. To reduce bias, the nomogram was constructed using a large-sample size cohort and validated internally. Second, we did not focus on patients with seropositivity for Musk or LRP4, who may also onset with ocular symptoms. This is mainly due to the low incidence of Musk or LRP4-MG and anti-Musk or LRP4 antibodies were not detected routinely in some neuromuscular centers. Third, although multivariable Cox regression models were used, residual confounding and confounding due to unmeasured factors cannot be ruled out. Fourth, the AUC is larger for the validation cohort than the development cohort. This is probably due to sample size and the high variability (as a result of the small sample size in the validation cohort). Meanwhile, this also reflected that the prediction model was not overfitted. Last, despite internal validation demonstrated good discrimination and calibration of the nomogram model, we did not perform external validation. Hence, external validation using other ethnic populations and centers with larger-scale sample size is warranted to improve the generalizability of the model.

## Conclusion

We developed a nomogram model that predicted 1- and 2-year generalization probabilities in patients with OMG and stratified these patients into distinct generalization risk groups. Based on five objective and easy-to-acquire variables, this nomogram and free web-based tool can aid neurologists in selecting suitable patients for initiating immunotherapy and for enrolment in clinical trials of risk-modifying treatments.

## Data Availability Statement

The data analyzed in the study are available from the corresponding authors on reasonable requests.

## Ethics Statement

The studies involving human participants were reviewed and approved by The Ethics Committee of Tangdu Hospital, The Fourth Military Medical University. Written informed consent from the participants’ legal guardian/next of kin was not required to participate in this study in accordance with the national legislation and the institutional requirements.

## Author Contributions

TC conceptualized the study, secured funding, and designed uniform procedures for data collection across the study centers. TC, ZL and ZR contributed to the study design. TC, ZR, CS, YLL, RG and FG contributed to the analysis and curation of data. TC, ZL and ZR contributed to the interpretation of data. ZR, CS, YLL, FG, RG, SW, QX, LY, TL, YL, MZ, YG, XL, HL, YT and TG contributed to the data collection. TC and ZR contributed to the drafting of the manuscript. All authors contributed to the article and approved the submitted version.

## Funding

The authors disclosed the receipt of the following financial support for the research, authorship, and publication of this article. This work was supported by the discipline innovation and development plan of Tangdu Hospital-major program of clinical research (Grant No. 2021LCYJ002), Key R & D plan of Shaanxi Province (Grant No. 2021ZDLSF02-01); National Key R & D Plan (Grant No. 2017YFC0907705); New clinical technology and new business Foundation of Tangdu Hospital (Grant No. XJSXYW2021008); Science and Technology Innovation Development Fund of Tangdu Hospital (Grant No. 2019QYTS006).

## Conflict of Interest

The authors declare that the research was conducted in the absence of any commercial or financial relationships that could be construed as a potential conflict of interest.

## Publisher’s Note

All claims expressed in this article are solely those of the authors and do not necessarily represent those of their affiliated organizations, or those of the publisher, the editors and the reviewers. Any product that may be evaluated in this article, or claim that may be made by its manufacturer, is not guaranteed or endorsed by the publisher.
