# Supplementary material for: Development and Validation of a Nomogram for Predicting Generalization in Patients With Ocular Myasthenia Gravis
Source: Front Immunol. 2022 Jul 7;13:895007. doi: 10.3389/fimmu.2022.895007 (PMC9302474; doi:10.3389/fimmu.2022.895007)
Supplement: Supplementary file 1 [file DataSheet_1.docx]

Supplementary Material

Supplementary Table 1. Ethics Committees of each medical center

| Master research center | Ethics Committee of Tangdu Hospital, the Fourth Military Medical University |
| --- | --- |
| Sub-centers | Ethics Committee of West China Hospital of Sichuan University |
|  | Ethics Committee of Henan Institute of Medical and Pharmaceutical Sciences |
|  | Ethics Committee of Jiangxi Provincial People's Hospital |
|  | Ethics Committee of Xianyang First People's Hospital |
|  | Ethics Committee of Xi’an No.1 Hospital |
|  | Ethics Committee of Xi'an Fourth Hospital |


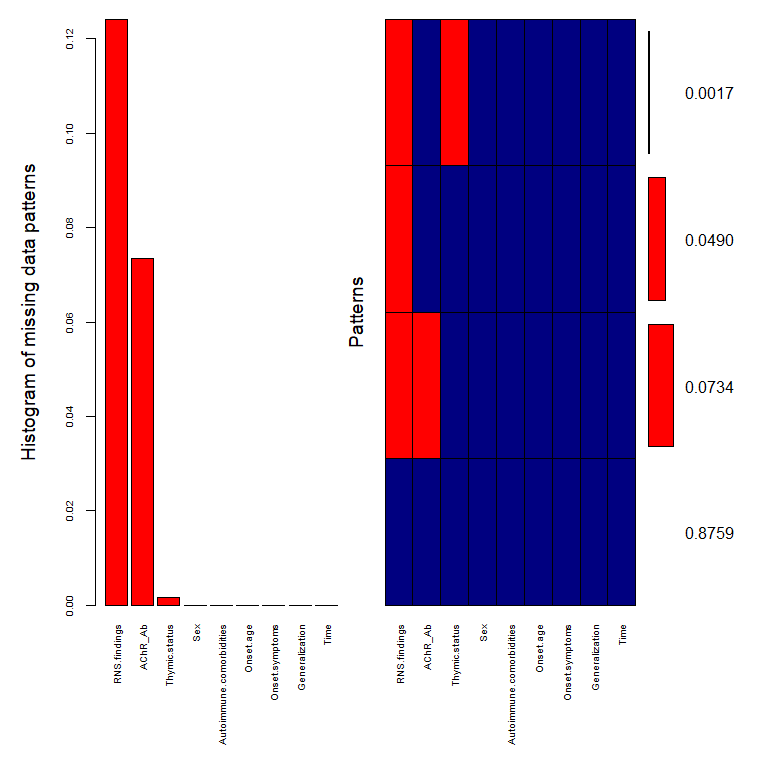


**Supplementary Figure 1.** **Histogram of missing value patterns**

The bar plot illustrates the missing proportion of variables with red bars, and the matrix diagram illustrates the proportion of missing patterns for different variables and the proportion of complete data. Red rectangles represent missing data, the blue rectangle represents the complete data.
